# The effect of organic acids and storage temperature on lite salad dressing rheology and *Zygosaccharomyces parabailii* growth

**DOI:** 10.1007/s13197-022-05459-4

**Published:** 2022-05-10

**Authors:** Alexander D. Meldrum, Gülhan Ünlü, Helen Joyner

**Affiliations:** 1 Department of Animal, Veterinary and Food Sciences, University of Idaho, Moscow, USA; 2 Food Product Development, Perfect Day, Inc., Berkeley, USA; 3 Department of Chemical and Biological Engineering, University of Idaho, Moscow, USA; 4 School of Food Science, Washington State University, Pullman, USA

**Keywords:** *Zygosaccharomyces parabailii*, Lite salad dressing, Spoilage by yeast, Rheology

## Abstract

*Zygosaccharomyces parabailii* (*Z. parabailii*) causes spoilage in salad dressings due to its tolerance to osmotic pressure. The objective of this study was to determine the effect of organic acids and storage temperatures (4, 10, and 25 °C) on *Z. parabailii* growth and salad dressing mechanical properties. Acetic, lactic, and gluconic acids were used alone and in combination to acidify salad dressing. *Z. parabailii*-challenged formulations containing acetic acid alone tended to have lower counts of *Z. parabailii* when compared to *Z. parabailii*-challenged formulations containing other acid combinations. Overall, storage temperature had the most impact on *Z. parabailii* growth over a 45-day storage. Acidulant type and combination impacted salad dressing mechanical properties. During the 45-day storage period, all formulations showed increased viscosity, a Herschel–Bulkley viscosity profile, and elastic-dominant viscoelastic behavior. The degree of change in rheological behaviors over time was dependent on the type of acid used in the formulation.

**Supplementary Information:**

The online version contains supplementary material available at 10.1007/s13197-022-05459-4.

## Introduction

A typical salad dressing is an oil-in-water emulsion with a formulation composed of oil, egg yolk, acidulants, and starch (FDA 2017b). Standard salad dressing formulations contain at least 30% fat by weight. The amount of fat in salad dressings may prompt health concerns for consumers, so lite salad dressings have been developed to make a healthier reduced-fat option. To be considered “lite,” a salad dressing with > 50% calories from fat needs a 50% fat reduction by weight from the original formulation. For a salad dressing with $\le$ 50% calories from fat to be considered “lite,” the final product needs a 33.33% reduction in all calories (Chiralt et al. 1992; US FDA 2017a). Modifying dressing formulas to meet lite specifications usually involves increasing starch and water content (Peressini et al. 1998). Additional starch and other hydrocolloids are needed to increase dressing emulsion stability. However, the increased starch levels cause lite dressing formulations to be susceptible to the growth of undesirable spoilage organisms, as starch is a potential nutritional source (Kurtzman et al. 1971). To prevent spoilage, salad dressing includes vinegar, lemon juice, or food grade acids. Most salad dressings are intended to be shelf-stable and require high levels of acids to keep microbes from growing at ambient temperatures. Typically, the antimicrobial properties of the acids in high acid foods will prevent the growth of spoilage organisms. Current industry practice is to use sorbic and benzoic acid to help prevent the growth of these spoilage microorganisms in salad dressings. However, certain spoilage organisms have higher levels of osmotic tolerance and can survive in an acidic environment. Thus, the use of these antimicrobials is not always effective, and growth of acid-tolerant organisms can result in early spoilage of the salad dressing.

A notable spoilage microorganism in salad dressings is *Zygosaccharomyces parabailii (Z. parabailii). Z. parabailii* is a yeast that shares similar microbiological characteristics and genetic traits with *Zygosaccharomyces bailii* (Suh 2013). As there are few published studies on *Z. parabailii*, many of the assumptions made about *Z. parabailii* are based on *Zygosaccharomyces bailii* metabolic and osmotic responses. *Z. parabailii’*s tolerance to low pH, salts, and antimicrobial compounds allows it to grow in salad dressings with little competition (Sousa-Dias 1996). It can grow in foods at pH between 2.0 and 7.0, as well as with up to 12.5% w/w salt content (Thomas and Davenport 1985). Spoilage from *Z. parabailii* has caused significant economic loss in the food industry (Smittle and Flowers 1982; Fleet 2007). Uncontrolled growth and fermentation will produce flavors, colors, and odors which are not palatable to consumers. Additionally, the rapid production of CO_2_ during fermentation can cause damage to packaging containers by creating high pressures in the bottle, potentially resulting in explosion of the container.

Chemical preservatives are commonly used to control the growth of pathogens and spoilage organisms in salad dressings (Warth 1977). These preservatives consist of antimicrobial compounds, such as ethylenediaminetetraacetic acid (EDTA), and organic acids, such as benzoic, sorbic, acetic, lactic, and gluconic acids (Stratford et al. 2013). As undissociated molecules, these organic acids rapidly diffuse through the microorganisms’ lipid bilayer; dissociation of these molecules within microbial cells releases protons (H^+^). This lowers the internal cell pH, inhibiting growth by causing highly extended lag phases. Unfortunately, organic acids are not as effective against acid-tolerant microorganisms such *Z. parabailii.* Several studies have investigated the mechanisms behind the high acid tolerance in *Z. parabailii* (Guerreiro et al. 2012; Stratford et al. 2013; Macpherson et al. 2005). The high resistance to weak acids was attributed to the ability of *Z. parabailii* to utilize organic acids and preservatives as carbon sources combined with the use of an H^+^ pump to remove H^+^ ions from within the cell (Macpherson et al. 2005). Because control of *Z. parabailli* with weak organic acids can be difficult and because the acid selected may impact salad dressing flow behaviors, the objective of this study was to determine the effects of using different combinations of organic acids and storage temperatures on *Z. parabailii* growth, as well as how these acid–temperature combinations affected salad dressing rheological properties during storage.

## Materials and methods

### Materials

Litehouse Inc. (Sandpoint, ID, USA) donated the ingredients to make the salad dressing including soybean oil (ADM, Cheney, WA, USA), enzyme modified egg yolk (Michael Foods, Minnetonka, MN, USA), MIRA-SPRESE (Tate & Lyle, Hoffman Estates, IL, USA), buttermilk powder (All American Foods, Inc, North Kingstown, RI, USA), sugar (National Sugar, Boise, ID, USA), maltodextrin (Tate & Lyle, Hoffman Estates, IL, USA), gum arabic (TIC gums, White March, MD, USA), Fastir xanthan (Tate & Lyle, Hoffman Estates, IL, USA), acetic acid (99.98% w/w Fischer, Hampton, NH), lactic acid (50% w/w Fischer, Hampton, NH), and gluconic acid (50% w/w Fischer, Hampton, NH). Lyophilized *Z. parabailii* (ATCC® 36,947™) was purchased from ATCC (Manassas, VA, USA). Tryptic glucose yeast extract agar (TGYE) and buffered peptone water were purchased from VWR (Radnor, PA, USA).

### Lite salad dressing preparation

Each formulation of lite salad dressing was prepared in a blender (Waring Commercial; Torrington, Connecticut, USA) at 8,000 rpm to promote formation of a stable emulsion. Water and water-soluble ingredients were first mixed together as follows (all amounts in w/w): 22.6% DI water, 21.4% sugar, 4.7% egg yolk, 2.6% buttermilk powder, 4.2% starch, 0.4% xanthan gum, 0.4% gum arabic, and 8.0% maltodextrin. After blending these ingredients for 30 s, 35.7% w/w soybean oil was added to the mixture with the blender running over a period of 60 s to create an oil-in-water emulsion. After emulsification, the formulations were acidified to a final pH, verified by a FiveEasy pH meter (Columbus, OH, USA), of either 3.2 or 4.2 based on the formulations in Table 1. The target pH of 4.2 was chosen based on a high-pH dressing made by a food manufacturer to evaluate the effectiveness of the weak acids on microbial growth in a worst-case scenario. The 3.2 pH target was selected based on preliminary research. Formulations followed a modified factorial design, with the goal of evaluating all acid alone and combined in at least a 1: 1 ratio. Acid combinations in 1: 2 ratios were selected based on preliminary studies. All formulations were made in triplicate. Each finished batch of salad dressing was separated into 500 mL lots and stored at 4, 10, or 25 °C. The lots were allowed to reach the desired storage temperature before inoculation with Z. *parabailii.*

**Table 1:** Food grade acid combinations used as potential mitigators of *Zygosaccharomyces parabailii* growth in lite salad dressing

Acidulant	Ratio	pH	% w/w	Abbreviations
Gluconic acid	–	4.2	1.00	GDL
		3.2	2.00	GDL2%
Acetic acid	–	4.2	0.50	AC
Lactic acid	–	4.2	0.50	LA
Gluconic + Lactic acid	1: 1	4.2	0.75	GL
	1: 2			GL2
Gluconic + Acetic acid	1: 1	4.2	0.75	GA
	1: 2			GA2
Gluconic + Acetic + Lactic acid	1: 1: 1	4.2	0.50	GAL
Acetic + Lactic acid	1: 1	4.2	0.75	AL

The formulations were compared to a dressing with the same formulation above prepared without the addition of an acidulant. This formulation spoiled before the first sample point for 10 and 25 °C, and had no significant changes at 4 °C. The control data was omitted from the reported data because all formulations with acidulants were able to prevent the growth better than a dressing without an acidulant, and the CFR standard of identity for salad dressing requires an acidulant to be used in the formulation (FDA, 2017b).

### Inoculation of salad dressings with *Z. parabailii*

An inoculum was prepared directly from *Z. parabailii* cells that were kept as frozen stocks at -80 °C. The 500 mL lots of previously prepared salad dressings were inoculated with *Z. parabailii* at a final concentration of approximately 1 × 10^4^ CFU/mL. The inoculated lots of salad dressing were distributed in 10 mL aliquots to 15 mL fermentation tubes (Company, State, USA). Inoculated 15 mL fermentation tubes were stored at 4, 10, or 25 °C for 45 days.

Three fermentation tubes from each batch were removed from storage every 5 days for plate counts and discarded after use. The salad dressing from the fermentation tubes was diluted (ten-fold serial dilutions) in buffered peptone water and the resulting dilutions were spread plated, in triplicate, on solid TGYE agar with 0.5% acetic acid for *Z. parabailii*. All microbiological analyses were conducted in a biological safety cabinet (NuAire, MN, USA). Inoculated plates were incubated at 25 °C and colonies were counted after 72 h.

### Small strain and rotational rheology

All rheological measurements were performed in triplicate on a DHR3 (TA Instruments; New Castle, Delaware, USA) with a cone and plate system (1° angle, 40 mm diameter) at 25 °C. Each sample was conditioned at 25 °C for 30 s, then presheared at 10 rad/s for 20 s. The sample was equilibrated for 60 s before the test started.

Shear rate sweeps were conducted from 0.01 to 100 1/s and 100 to 0.01 1/s to evaluate viscosity profiles and hysteresis. Strain sweeps were conducted from 0.01 to 100% strain at 1 rad/s to determine critical strain and parameter values at critical strain. Critical strain and stress, or the strain and stress at the end of the linear viscoelastic region (LVR) were determined as the strain–stress pair at which the complex modulus deviated from the previous value by more than 3%. Frequency sweeps were performed at 75% of the smallest critical strain for all formulations to evaluate small-strain viscoelastic behaviors. Frequency sweeps were conducted at 0.0775% strain from 0.1 to 100 rad/s.

### Data analyses

Microbial counts were analyzed for statistical differences with SAS software version 9.1 (SAS, Cary, NC) using a two-way analysis of variance (ANOVA) followed by Tukey’s test. Significant differences were recorded at *P* < 0.05. Viscosity curves for each formulation were averaged together and the resulting average curves fitted to a Herschel Bulkley model, $\sigma = \sigma_{o} + K\dot{\gamma }^{n}$, where shear stress, consistency coefficient, shear rate, flow behavior index, and yield stress are represented by $\sigma$ (Pa), $K$ (Pa.s^n^), $\dot{\gamma }$ (1/s), $n$, and $\sigma_{o}$ (Pa) respectively.

## Results and discussion

### Effects of organic acids on *Z. parabailii* growth in lite salad dressings

The type of acid used resulted in significant differences in the concentration of *Z. parabailii* in different formulations of salad dressing stored at 25 °C (Table 1). Comparing formulations with a single acidulant, AC had the lowest concentration at 2.85 × 10^6^ ± 0.6 of *Z. parabailii* after 45 d of storage, while GDL at 1.26 × 10^7^ ± 0.01 and GDL2% 1.22 × 10^7^ ± 0.03 had the highest concentration of *Z. parabailii* after 45 d of storage*.* It should be noted that although the use of different acids led to varying rates of growth and level of counts over time, *Z. parabailii* counts in all formulations reached 10^5^ CFU/mL by Day 5. At this concentration of *Z. parabailii*, salad dressing develops off-flavors and other noticeable spoilage characteristics, such as gas production, due to fermentation (Kurtzman and Fell  2006). All formulations at 25 °C, regardless of acidulant or acidulant combination, showed gas production after 5 d of storage, indicating spoilage.

**Fig. 1:**
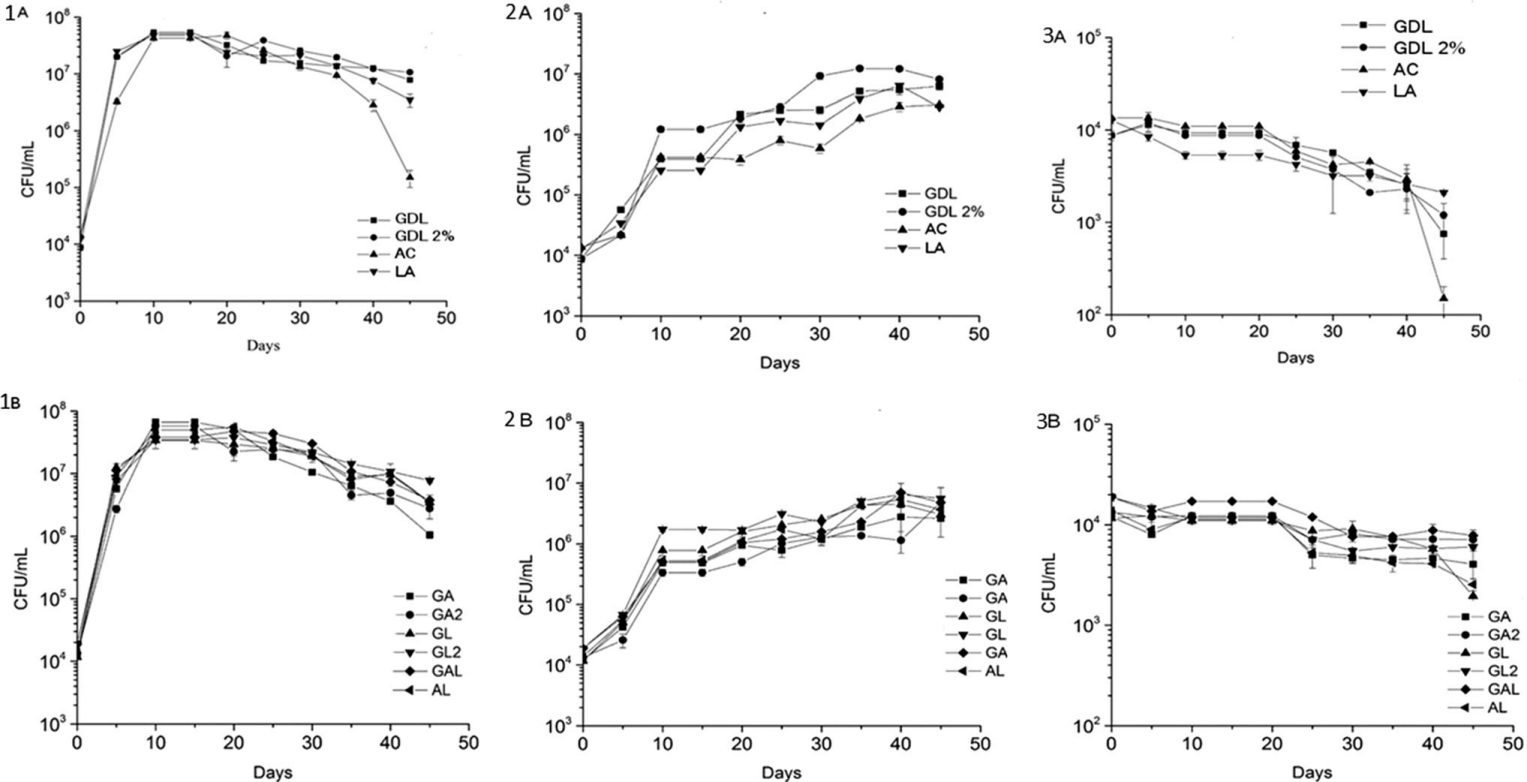
*Z. parabailii* growth in lite salad dressings prepared with **a** individual acids and **b** combinations of acids over a 45-day storage period at 25 °C (1), 10 °C (2), and 4 °C (3). Error bars represent standard error

Comparing formulations stored at 25 °C with multiple acidulants, GA at 3.65 × 10^6^ ± 0.5 formulation had the lowest concentration of *Z. parabailii* and GL2 at 1.08 × 10^7^ ± 0.35 had the highest concentration of *Z. parabailii* after 45 d of storage. Similar to the single-acidulant formulations, all *Z. parabailii-*challenged formulations with multiple acidulants had rapid yeast growth during the first several days of storage, which resulted in dressings with concentrations of *Z. parabailii* over 10^5^ CFU/mL after 5 d of storage (Fig. 1 (1)). Differences in *Z. parabailii* growth among formulations may have been related to differences in the chemical properties of the weak organic acids used in the formulation. Each organic acid used in this study varied in molecular weight, hydrophobic/hydrophilic properties, pKa, and other chemical properties. These differences, particularly differences in pKa (Lambert and Stratford 1999), would have impacted the degree to which each weak acid could affect the cellular pH of *Z. parabailii.*

Overall, the concentration of any weak organic acid used was not high enough to inhibit the growth of *Z. parabailii* at 25 °C (Fig. 1 (1)). Interestingly, the GDL2% formulation did not have the expected impact on reduction of *Z. parabailii* at 25 °C when compared to GDL (1% gluconic acid). The higher concentration and lower pH of GDL2% were expected to have a greater inhibitory effect on *Z. parabailii* growth but GDL and GDL2% had no significant differences in terms of *Z. parabailii* growth (Table S1). Thus, the concentration of gluconic acid must be greater than 2% to have an inhibitory effect on *Z. parabailii*.

Weak acid can damage yeast cells by entering the cytoplasm of the cell through simple diffusion (Warth 1989; Stratford et al. 2013). The rate of diffusion of a weak acid into the cell usually starts rapidly but slows to an equilibrium rate over time. Along with simple diffusion, weak acids have their own pH-dependent equilibrium that causes the weak acid to dissociate. The rate and degree of dissociation is related to the pKa of the weak acid. Since the internal pH in the cytoplasm is typically higher than the environment, weak acids dissociate to their conjugate acids and bases in the cytoplasm. This disassociation can lead to a reduction in the cytoplasm pH, which can cause damage to key enzymes used for *Z. parabailii* cellular function (Stratford et al. 2013). Damage to the enzymes can stop the enzyme from functioning, leading to cell death. However, complete inhibition during the 45-day storage period was not observed in samples stored at 25 °C. This may be due to *Z. parabailii* resistance to dissociation of weak organic acids in its cytoplasm. One mechanism through which *Z. parabailii* may have maintained its internal pH is metabolism of the dissociated forms of different organic acids used in the salad dressing formulations. Other studies have shown that acetate and glucono delta-lactone, the dissociated forms of acetic acid and gluconic acid, respectively, can be catabolized through the TCA cycle in *Z. parabailii* cells (Guerreiro et al. 2012; Macpherson et al. 2005). Another possible mechanism for defense against weak organic acids is the ability of *Z. parabailii* to pump H^+^ ions out of the cell through its H^+^–ATPase pumps (Macpherson et al. 2005), which would prevent cell pH from dropping too low to support life.

The rate of *Z. parabailii* growth at 10 °C was significantly lower than the rate of growth at 25 °C (Fig. 1. (1)(2)). This change in growth rate from 25 °C was probably due to the decrease in the rate which enzymes used for cellular respiration and replication could react in the cytoplasm at lower temperatures (Kurtzman et al. 2011). However, no formulation was able to completely inhibit the growth of *Z. parabailii* at 10 °C: all samples reached concentrations of *Z. parabailii* ≥ 10^5^ CFU/mL between 5 and 10 days of storage, indicating spoilage*.* The type of acid used in the formulation resulted in significant differences in *Z. parabailii* concentration in formulations stored at 10 °C (Table S1). When the concentration of *Z. parabailii* was < 10^5^, AC and GDL2% formulations had the lowest concentration of *Z. parabailii* (Fig. 1. (2)a))*.* Before the formulations with a combination of acidulants reached *Z. parabailii* concentrations > 10^5^ CFU/mL (i.e. during the first few days of storage), GA had the lowest concentration of *Z. parabailii* (Fig. 1. (2)a). As the concentration of *Z. parabailii* increased in the formulations stored at 10 °C, the ability of organic acids to affect the growth of *Z. parabailii* altered. At the end of the 45-day storage period, formulations containing acetic acid generally had lower concentrations of *Z. parabailii* compared to formulations that did not. Overall, GDL and GDL2% had higher concentrations of *Z. parabailii* over time compared to the other formulations (Fig. 1. (2)). These results agreed with the growth observed at 25 °C. *Z. parabailii* has been shown to have the ability to assimilate and metabolize d-glucono-1,5-lactone (Suh et al. 2013). Therefore, the increased growth of *Z. parabailii* in formulations containing gluconic acid can be attributed to its ability to ferment the cyclic ester of gluconic acid, glucono delta-lactone.

Salad dressings stored at 4 °C were the only samples to show inhibitory effects on *Z. parabailii* growth*.* All formulations showed downward trends of *Z. parabailii* counts starting around 20 d of storage (Fig. 1. (3)). For the first 20 d, GAL had the highest concentration of *Z. parabailii*, LA had the lowest, and all the other formulations showed no significant differences in concentration (Table S1). After 45 d, AC had the most impact on *Z. parabailii* growth, as it reduced the concentration of *Z. parabailii* by 2 logs. GDL and GDL2% reduced the concentration by approximately 1 log after 45 d, and the other formulations reduced the concentration of *Z. parabailii* growth by approximately 0.5 log*.*

In general, temperature played a critical role in the growth of *Z. parabailii*. For any microorganism, the temperature range suitable for growth is dictated by enzyme kinetics and the ability of molecules to move through the cytoplasm. At lower temperatures (e.g. 4 °C), *Z. parabailii* cellular respiration could be affected by low enzyme activity and reduced availability of other molecules within the cell. *Z. parabailii* was best inhibited at 4 °C as all formulations used in this study had concentrations of *Z. parabailii* below 10^5^ CFU/mL. An elevation in temperature from 4 to 10 °C was adequate for *Z. parabailii* to proliferate to spoilage levels after only a few days of storage. Thus, 4 °C seemed to be the threshold temperature for inhibition of *Z. parabailii* growth and prevention of growth-induced spoilage.

### Viscosity of lite salad dressing with multiple acidulants

All lite salad dressing formulations showed Herschel–Bulkley behavior (Table 2). Herschel–Bulkley fluids show both shear-dependent behavior and a yield stress (Peressini et al. 1998). All samples showed shear-thinning behavior ($n < 1$) across all days (Table 2). However, each formulation had significant differences in consistency coefficient, yield stress, and extent of shear thinning (Table 2). The differences in the viscosities can be attributed to the hydrophobic/hydrophilic properties of the weak acids used in the formulations. Both the ability for acids to interact at the oil/water interface and effects of the different pKa of the acids on the starch may have resulted in changes to the viscosity behaviors (Romero et al. 2009).

**Table 2:** Viscosity profiles, where $\sigma = \sigma_{o} + K\dot{\gamma }^{n}$, for low-calorie salad dressings

Sample	*N*	*K* (n Pa∙sn)	${\varvec{\sigma}}_{{\varvec{o}}}$(Pa)	Sample	*N*	*K* (n Pa∙sn)	${\varvec{\sigma}}_{{\varvec{o}}}$(Pa)
*Day 0*				*Day 15*			
GDL	0.46	22.2 ± 0.6	14.7 ± 0.3	GDL	0.46	22.6 ± 0.6	16.8 ± 0.5
AC	0.32	16.1 ± 0.1	13.2 ± 0.4	AC	0.32	18.7 ± 2.4	15.4 ± 2.7
LA	0.46	21.1 ± 0.6	15.4 ± 0.3	LA	0.47	19.9 ± 1.2	17.0 ± 2.2
GA	0.42	19.6 ± 0.8	17.2 ± 0.4	GA	0.45	22.0 ± 2.1	18.4 ± 0.8
GA2	0.44	19.2 ± 0.6	15.4 ± 0.2	GA2	0.48	20.3 ± 0.2	16.1 ± 0.3
GL	0.48	18.5 ± 0.5	18.0 ± 0.3	GL	0.49	20.9 ± 0.4	18.0 ± 0.7
GAL	0.46	17.0 ± 0.5	15.4 ± 0.3	GAL	0.49	18.6 ± 0.6	21.0 ± 0.6
AL	0.49	16.4 ± 0.1	16.6 ± 0.1	AL	0.51	17.3 ± 0.6	17.2 ± 1.8
*Day 30*				*Day 45*			
GDL	0.47	28.2 ± 0.2	22.1 ± 1.2	GDL	0.46	29.7 ± 0.13	23.3 ± 1.4
AC	0.47	26.8 ± 2.0	15.2 ± 1.3	AC	0.45	27.2 ± 2.2	18.3 ± 1.8
LA	0.53	22.4 ± 0.3	20.3 ± 0.6	LA	0.55	24.0 ± 0.4	24.0 ± 0.2
GA	0.47	24.3 ± 0.7	25.1 ± 1.0	GA	0.52	24.4 ± 1.8	29.9 ± 1.4
GA2	0.49	26.9 ± 0.6	30.6 ± 1.7	GA2	0.47	20.9 ± 1.2	17.1 ± 0.7
GL	0.50	25.1 ± 0.5	28.2 ± 0.2	GL	0.37	26.8 ± 0.6	31.2 ± 0.6
GAL	0.51	19.5 ± 0.2	22.0 ± 0.5	GAL	0.52	21.1 ± 0.1	24.0 ± 3.2
AL	0.53	19.7 ± 0.4	17.4 ± 0.1	AL	0.53	19.5 ± 2.1	17.7 ± 0.1

The consistency coefficient, $K$ (Pa.s^n^), has a strong relationship with viscosity. GDL formulation had the highest $K$ value for all timepoints. Gluconic acid can interfere with electrostatic interactions by influencing the charges on the polysaccharide. The degree to which any weak acid can affect the charges on polysaccharides can be associated with its pKa. Gluconic acid has a pKa of 3.86, which is lower than the pKa of acetic acid (pKa = 4.76) but equal to the pKa of lactic acid. Both GDL and LA had similar viscosity parameters on Day 0. Therefore, the differences between formulations were attributed to the differences in the pKas of the acids used (Hamdine et al. 2005). For individual acids, $K$ decreased as pKa increased. AC, which had the lowest $K$, $n$, and $\sigma_{o}$, had the highest pKa.

When the acids were used in combination, their effects on viscosity were determined by the particular combination of acids. GA, GA2, GAL, GL, and AL had higher $K$ and $n$ values than AC, but smaller $K$ and $n$ values than GDL and LA on Day 0. The presence of acetic acid had a larger effect on $K$ and $n$ values on Day 0 for formulations containing multiple acidulants as compared to the presence of gluconic or lactic acid. The presence of multiple acids seemed to have a synergistic effect on $\sigma_{o}$, as those values were all higher compared to formulations with a sole acidulant on Day 0. Yield stress represents the amount of stress that is needed for a material to flow; higher yield stress indicates more energy is needed to break down the internal structure and induce flow (Steffe 1996). The combination and dissociation of different acids used in AG, AG2, AL, GAL, and AL may have altered the charge distribution in the salad dressing structure, leading to an increase in van der Waals forces between polysaccharides and proteins. This in turn would lead to increased yield stress for salad dressings formulations with acid combinations.

The *K* values increased over time for all formulations. Starches and hydrocolloids create internal structures during quiescent storage through entanglement of long polysaccharide chains, which would cause an increase in $K$ over time (Felix et al. 2017). These internal forces can create resistance to flow, subsequently increasing $K$ and $\sigma_{o}$. The amount of change in the $K$ values was influenced by the specific acid(s) used. For example, AC had a 70.0% increase in $K$ over 45 days compared to the 13.7% increase in *K* in LA (Table 2). Lactic, gluconic, and acetic acid may be affecting the degree of polysaccharide rearrangement in the formulations over time. Other than a general increase in $K$ values, the particular acidulant(s) used did not have any definitive trends over time. The $K$ values were expected to increase during storage as starches, gums, and proteins undergo structural rearrangements over time, resulting in a higher consistency coefficient.

Both $n$ and $\sigma_{o}$ values also increased significantly over time for all samples, indicating reduced shear-thinning behavior and increased yield stress over time. The decrease in shear-thinning behavior over time may have been related to breakdown of the polysaccharide microstructures in the salad dressing formulation. The increased yield stress was likely due to an increase in van der Waals forces and increased polysaccharide entanglement over time, requiring more energy to induce flow. Samples containing an equal ratio of gluconic acid plus another acidulant had greater increases in $\sigma_{o}$ over time compared to the other samples. The equal ratio of gluconic acid to acetic or lactic acid possibly had a greater effect on the charge distribution between polysaccharides over time. The pKas of acetic acid and gluconic acid are not equal; this might affect charge distribution between polysaccharides and proteins. A lower net charge would increase the electrostatic interaction between polysaccharides, which in turn increased $\sigma_{o}$ values (Table 2).

Overall, acid type had a notable impact on $K$, $\sigma_{o}$, and $n$ values. Viscosity profiles give foods a certain mouthfeel; changes in salad dressing viscosity profiles may lead to textural differences (Liu et al. 2007). However, differences in the $K$, $\sigma_{o}$, and $n$ values among the formulations were small and may not have created noticeable texture differences. Sensory studies such as descriptive analysis would need to be conducted to provide conclusive textural data.

### Strain sweep for lite salad dressings composed of different acids

Strain sweep data were used to calculate critical strains to ensure that frequency sweeps were conducted within the linear viscoelastic region (LVR) (Franco et al. 1997). All samples exhibited elastic-dominated behavior at critical strain (Table 3). The elastic modulus $G^{\prime}$ (Pa), loss modulus $G^{\prime\prime}$ (Pa), complex modulus $G^{*}$(Pa), and phase angle (degrees) values of most samples significantly decreased during the 45-day storage period. The only exception was GL, which had a significant increase in $G^{\prime}$ values over the 45-day storage period. No differences were found among the critical strain values for all formulations; thus, all formulations showed similar LVRs. The critical strains of all formulations did not show significant differences between Day 0 and Day 45. This was not expected based on the viscosity results. The viscosity curves showed an increase in the yield stress over time, so it was expected that the critical strain values would similarly increase. A possible explanation for this may be that the yield stress changes over time as measured by shear rate sweeps were relatively small and had insignificant impact on critical strain. The decrease in phase angle over time indicated that samples had increased elastic-dominated behaviors with increased storage time. This behavioral change was attributed to the rearrangement of polysaccharides in the salad dressing formulations. Polysaccharide entanglement in the dressing could have given additional structure to the salad dressing, causing an increase in elastic-type behavior (Santiago et al. 2002).

**Table 3:** Critical values from strain sweep for formulations stored for 0 and 45 days^a^

Day	Sample	G′ (Pa)	G″ (Pa)	Strain (%)	G* (Pa)	Phase angle (deg)
0	GDL	255^B^	64.2^D^	0.251^A^	262^CB^	14.1^F^
	AC	236^C^	67.8^DC^	0.250^A^	245^CD^	16.0^A^
	LA	250^B^	66.1^DC^	0.250^A^	259^CB^	14.7^E^
	GA	294^A^	80.8^AB^	0.249^A^	304^A^	15.3^D^
	GA2	253^B^	71.6^BC^	0.251^A^	263^B^	15.7^BC^
	GL	221^DC^	60.6^D^	0.251^A^	229^ED^	15.3^D^
	GL2	283^A^	81.7^A^	0.250^A^	295^A^	16.0^A^
	GAL	216^D^	61.4^D^	0.251^A^	224^E^	15.8^AB^
	AL	189^E^	53.3^E^	0.251^A^	196^F^	15.7^C^
45	GDL	270^A^	62.2^A^	0.251^A^	277^A^	12.9^ED^
	AC	165^DC^	43.8^BC^	0.250^A^	171^ED^	14.8^A^
	LA	202^B^	46.1^BC^	0.250^A^	207^C^	12.8^E^
	GA	212^B^	49.4^BC^	0.251^A^	218^C^	13.1^D^
	GA2	175^C^	44.4^BC^	0.250^A^	181^D^	14.2^C^
	GL	254^A^	56.9^AB^	0.250^A^	261^B^	12.6^F^
	GL2	210^B^	48.7^BC^	0.250^A^	216^C^	13.0^D^
	GAL	137^E^	34.4^C^	0.250^A^	141^F^	14.0^C^
	AL	151^D^	38.9^BC^	0.250^A^	156^E^	14.4^B^

^a^For each day, letters in each column that are different indicate significant differences (*p* < 0.05)

$G^{*}$ values generally decreased over time. The differences in $G^{\prime}$, $G^{\prime\prime}$, and $G^{*}$ values among the formulations, although statistically significant, were slight and may not result in noticeable differences in terms of processing behavior or textural attributes. As previously suggested, sensory studies are needed to determine whether differences in moduli result in different texture perceptions.

### Frequency sweeps for lite salad dressings formulated with different acidulants

**Fig. 2:**
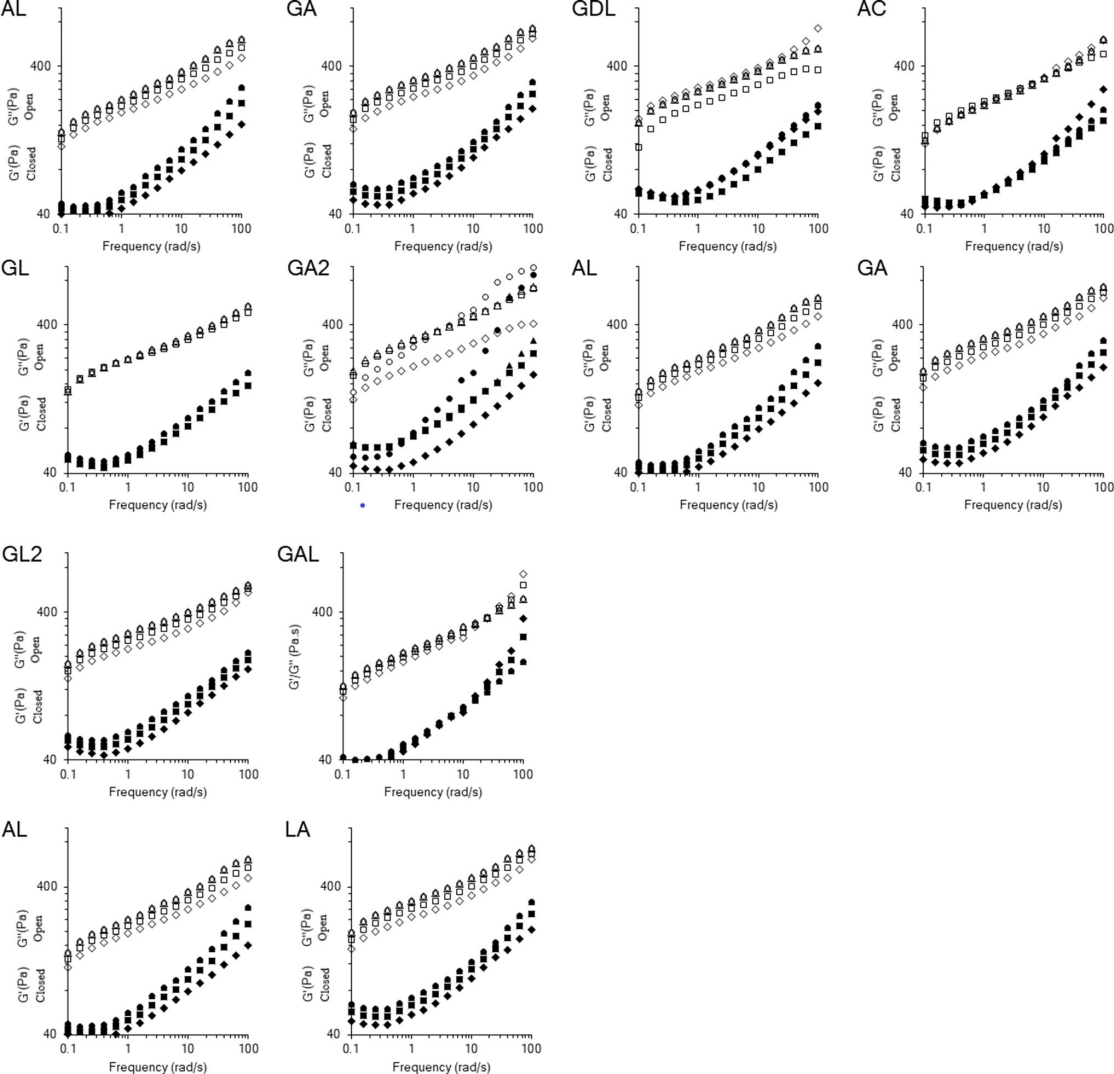
Frequency sweep for lite salad dressing formulations made with different acid combinations. Formulations were tested on Day 0 (○), Day 15 (△), Day 30 (□), and Day 45 (♢). Open symbols represent $G^{\prime}$ closed black symbols represent $G^{\prime\prime}$

All formulations showed weak gel viscoelastic behavior (Fig. 2.). A weak gel is an intermediate between a solid and a liquid which shows mechanical rigidity. The gel in the salad dressing consisted of polysaccharide polymers, which entangle in the aqueous phase of the dressing giving it a network structure and a yield stress (Saha and Bhattacharya 2010). A weak gel is indicated by $G^{\prime} > G^{\prime\prime}$ combined with frequency dependence as indicated by an increase in $G^{\prime}$ with frequency. All formulations showed weak gel and elastic-dominated behavior ($G^{\prime} > G^{\prime\prime}$), which aligned with the strain sweep data. Many of the formulations showed a decrease in both the storage and loss modulus values after 45 d (Fig. 2). The decrease in these values did not necessarily indicate a decrease in elastic behavior; rather, the decrease in the moduli vales were associated with the ability of the formulation to store and dissipate energy (Vianna-Filho et al. 2013). Over time, the carboxyl groups of the polysaccharide chains become ionized by the H^+^ ions and conjugate bases of the dissociated weak acids in the food system. Polysaccharide ionization may affect the way it interacts with the lecithin in the egg yolks, which in turn affects the macroscopic properties of the salad dressing (Myers, 1990).

Polysaccharides interact with themselves through physical association of their polymer chains, hydrogen bonding, hydrophobic association, and cation mediated cross-linking (Saha and Bhattacharya 2010). The ability for polysaccharides to interact with emulsifying agents, other polysaccharides, and themselves was impacted by the acids used in formulation. For example, AC and GL, unlike the other formulations, showed no significant changes in $G^{\prime}$ and $G^{\prime\prime}$ over the 45-day storage period. Most formulas showed a decrease in moduli values over the 45-day storage period. GDL moduli values, on the other hand, were statistically similar on Day 0 and Day 15, significantly decreased at 30 d of storage but increased after 45 d of storage. The increase in moduli values for GDL at Day 45 may have been due cross-linking of long polymer chains in the dressing formulation. The crosslinked chains may have difficulty in sliding past each other, resulting in a greater extent of elastic-type behavior.

In general, different acid combinations significantly impacted the viscosity, small-strain, and inhibition of *Z. parabailii* growth*.* All formulations used in this study were unable to stop the growth of *Z. parabailii* at 25° and 10 °C during the 45-day storage period, but the formulation that had the lowest concentrations of *Z. parabailii* at the end of the 45-day storage at either of these temperatures was AC. AC samples, which had acetic acid as a sole acidulant, also had the lowest counts of *Z. parabailii* in 4 °C. Changes in the viscoelastic properties and viscosities in all formulations were slight and may not affect sensory attributes. While sensory studies would need to be conducted for conclusive information on perceived textures, GA2 had the least changes in viscosity behavior over time, and GL had the least changes in viscoelastic properties over time, indicating better formulation stability during storage compared to the other formulations. However, these two formulations were not as effective as AC at reducing *Z. parabailii* counts*.* The use of acetic acid as a sole acidulant may not yield formulations with the same stability over time as other combination of acidulants used, but the ability of acetic acid to inhibit growth of *Z. parabailii* more effectively makes it a better choice of acidulant when formulating salad dressing for *Z. parabailii* growth.

## Conclusion

Using various combinations of weak acids in lite salad dressing resulted in significant differences in *Z. parabailii* concentration over a 45-day storage period. However, storage temperature had a notably larger effect on the growth of *Z. parabailii* than the type of acidulant(s) used. Of the organic acids evaluated in this study, acetic acid was the most effective for reducing *Z. parabailii* growth. Although the use of organic acids did not stop *Z. parabailii* growth at 10 or 25 °C, combining acidulant use with use of refrigeration temperatures (4 °C or lower) inhibited the growth *Z. parabailii* over 45 d of storage. Even though *Z. parabailii* growth rates were significantly reduced at temperatures around 10 °C as compared to those at 25 °C, dressings formulations still showed counts higher than 10^5^ CFU/mL at 10 °C, which indicated spoilage*.* Viscoelastic behaviors and changes to these behaviors over time were dependent on the acids used in the formulation. Changes in salad dressing acid composition impacted viscosity and viscoelastic properties. However, the differences in rheological behavior between formulations were small and may not yield a significant difference in textural perception. Overall, acetic acid was considered the most effective acidulant of the acids studied, even though GA2 and GL formulations showed a lesser degree of rheological changes over time. The most effective method of reducing *Z. parabailii* counts in salad dressing was by storing the dressing at 4 °C with acetic acid as an acidulant.

## Supplementary Information

Below is the link to the electronic supplementary material.

Supplementary file1 (DOCX 20 KB)

## Data Availability

All data generated or analyzed during this study are included in this published article [and its supplementary information files].

## Abbreviations

*Z. parabailii*: *Zygosaccharomyces bailii*
CFR: Code of federal regulations
TGYE: Tryptone glucose yeast extract agar
LVR: Linear viscoelastic region
RPM: Rotations per minute
*σ*: Shear stress
*K*: Consistency coefficient
$$\dot{\gamma }$$: Shear rate
*n*: Flow behavior index
*σ*_*o*_: Yield stress
*G*^′^: Elastic modulus
*G*^″^: Loss modulus
*G*^*^: Complex modulus
