# Supplementary material for: The effect of organic acids and storage temperature on lite salad dressing rheology and Zygosaccharomyces parabailii growth
Source: J Food Sci Technol. 2022 May 10;59(10):4075–84. doi: 10.1007/s13197-022-05459-4 (PMC9525512; doi:10.1007/s13197-022-05459-4)
Supplement: Supplementary file 1 — Supplementary file1 (DOCX 20 KB) [file 13197_2022_5459_MOESM1_ESM.docx]

**Supplemental Table:**

**Table S1** Tukey’s grouping for *Z. parabailii* growth in salad dressings formulations stored at 4°C, 10°C, and 25°C^a^

| Formulations | Tukey grouping 25°C | Tukey Grouping 10°C | Tukey Grouping 4°C |
| --- | --- | --- | --- |
| GDL | A | CB | CD |
| GDL 2% | ABC | A | D |
| A | BCD | E | CB |
| L | ABCD | C | D |
| GA | AB | ED | CB |
| GA2 | ABCD | E | B |
| GL | CD | CB | B |
| GL2 | D | B | B |
| GAL | ABCD | CD | A |
| AL | AB | C | CB |

^a^ Different letters in different columns indicate significant differences (α = 0.05)
